# Reference standard to read the air-driven calloric reflex test results

**DOI:** 10.1016/S1808-8694(15)30824-7

**Published:** 2015-10-18

**Authors:** Mauricio Malavasi Ganança, Marco Aurélio Bottino, Roseli Saraiva Moreira Bittar, Heloisa Helena Caovilla, Fernando Freitas Ganança

**Affiliations:** 1 MD. Full Professor of Otorhinolaryngology of the Federal University of São Paulo Medical School - Universidade Federal de São Paulo – Escola Paulista de Medicina (UNIFESP-EPM). Professor at the Graduate Program of Vestibular Rehabilitation and Social Inclusion - Universidade Bandeirante de São Paulo (UNIBAN).; 2 MD. PhD. Assistant Professor of Neurotology – University of São Paulo Medical School Teaching Hospital - Hospital das Clínicas da FMUSP.; 3 MD. PhD. Assistant Professor – Department of Neurotology - University of São Paulo Medical School Teaching Hospital - Hospital das Clínicas da FMUSP.; 4 Speech and Hearing Therapist. Associate Professor – Department of Otology and Neurotology - Federal University of São Paulo Medical School - Universidade Federal de São Paulo – Escola Paulista de Medicina (UNIFESP-EPM).; 5 MD. Adjunct Professor – Otology and Neurotology Course - Federal University of São Paulo Medical School - Universidade Federal de São Paulo – Escola Paulista de Medicina (UNIFESP-EPM). Professor at the Graduate Program of Vestibular Rehabilitation and Social Inclusion - Universidade Bandeirante de São Paulo (UNIBAN)..

The caloric reflex test is one of the most important procedures in the functional diagnosis of the vestibular system, because it allows for an independent evaluation of the right and left ears, identifying the labyrinth side (s) involved and characterizing the disorder intensity. Similarly to other tests used for a functional investigation of the vestibular system, the caloric reflex test has great intra and inter-individual variability regarding the values obtained.

The thermal stimulation of the labyrinth produces convection currents in the endolymph within the lateral semicircular canal being examined. As a consequence, an endolymphatic current is generated and its direction is based on the temperature used, whether warmer or colder than the endolymph. The endolymphatic current bend the sensorial cells of the lateral canal ampullar crest, triggering the ocular-vestibular reflex, which can be observed as a nystagmus. The cold test produces an ampullifugal current, which inhibits the sensorial structures and creates the nystagmus in the opposite direction of the labyrinth being tested; the warm test creates an ampullipetal current, which excites the sensorial structures and causes nystagmus in the same direction of the labyrinth being investigated. Besides the convection current, the mechanical transduction of the cupula, the thermal effect on the sensorineural structures, the central or neural adaptation and the non-linear combination of these mechanisms could be involved in the generation of the post-caloric nystagmus.

Throughout the years, water, air, ether and ethyl chloride have been used to stimulate the labyrinth by means of the caloric reflex test. Such test with cold and warm water is the most stimulating method, and also the one most employed. The air-driven caloric test is more comfortable for the patient and has the advantage that it can be performed when the patient has a tympanic membrane perforation, otitis externa or otitis media.

Many types of caloric tests have been advocated: the alternate bithermal labyrinthine stimulations, simultaneous bithermal, monothermal and tests with cold. The most employed method is the alternate bithermal stimulation, with equidistant temperatures from the body temperature (37°C). The flushing water must be 7°C above (warm test) and 7°C below (cold test) the body temperature. For the warm test, we use 44°C and for the cold one, the water must be 30°C. In order to produce response with an intensity similar to those achieved with water stimulation, the air temperature must be 13°C above and 13°C below body temperature, corresponding to 50°C for the warm test and 24°C for the cold one.

Results from the caloric reflex test can be recorded and analyzed by different procedures (electronystagmography, vector nystagmography or videonystagmography), with or without computerized interpretation. The most important parameter to use for the quantitative evaluation of the caloric test with water or air is the maximum angular velocity of the slow nystagmus component, which is proportional to the intensity of the stimulus employed. Studies have shown that the test-retest results and responses with air and water are similar and adequate for clinical use. The caloric reflex test interpretation must take into account the conditions in which it was carried out, because the results suffer direct influence of the type of stimulation as well as the equipment used.

Professors from the Federal University of São Paulo - Escola Paulista de Medicina (UNIFESP) and from the University of São Paulo (USP), within a research partnership agreement between these two institutions are working together, aiming at studying the results of the air-driven caloric test in healthy individuals, with the goal of establishing a reference standard for its clinical use in our settings. This standardization study has the participation of the following institutions: Pontifícia Universidade Católica de Campinas (PUCCAMP), Universidade Estadual de Campinas (UNICAMP), Universidade de Brasília (UNB), Universidade do Vale do Sapucaí (UNIVAS) and the Universidade Bandeirante de São Paulo (UNIBAN). The results from such study will be soon published in the Brazilian Journal of Otorhinolaryngology.

